# Clinical prediction model by machine learning to determine the results of maternal dietary avoidance in food protein-induced allergic proctocolitis infants

**DOI:** 10.3389/fped.2025.1612076

**Published:** 2025-05-16

**Authors:** Jing Li, Meng-yao Zhou, Yang Li, Xue Wu, Xin Li, Xiao-li Xie, Li-jing Xiong

**Affiliations:** Department of Pediatric Gastroenterology, Chengdu Women’s and Children’s Central Hospital, School of Medicine, University of Electronic Science and Technology of China, Chengdu, China

**Keywords:** food protein-induced allergic proctocolitis, breastfeeding, hypoallergenic formula, prediction model, dietary avoidance

## Abstract

**Objective:**

The objective of this study was to investigate the factors associated with the results of maternal dietary avoidance in infants diagnosed with Food Protein-Induced Allergic Proctocolitis (FPIAP). Additionally, we aimed to develop a predictive model using machine learning techniques to forecast the results of maternal dietary restrictions.

**Methods:**

The clinical data of FPIAP infants were retrospectively analyzed. The FPIAP infants were divided into two groups based on the results of maternal dietary restriction, and an analysis was conducted to identify the influencing factors. Variable was selected by Lasso regression model. Classification models were built utilizing various machine learning algorithms including XGB Classifier, Logistic Regression, Random Forest Classifier, Ada Boost Classifier, KNeighbors Classifier, LGBM Classifier, Decision Tree Classifier, Gradient Boosting Classifier, Support Vector Classifier. The optimal algorithm was selected to construct the final prediction model.

**Results:**

In a retrospective cohort study of 693 children diagnosed with FPIAP, the remission rate associated with maternal dietary avoidance was 47.38%. The overall efficacy of hypoallergenic formula was 88.48%. Multivariate analysis identified several factors influencing the outcome of maternal dietary restriction, including age, disease duration, regurgitation, eczema, and neonatal history of hematochezia. Variables were selected and incorporated into multiple machine learning models. Among them, the logistic regression model demonstrated relatively high stability and was ultimately selected for modeling. The final model achieved an AUC of 0.743 in the test set and an accuracy of 0.699. The validation set's AUC was within 10% of the test set's value, indicating acceptable generalizability. The Hosmer-Lemeshow goodness-of-fit test confirmed that the logistic regression model fit the data well (*P* = 0.691 > 0.05). Finally, a nomogram was used to visualize the model's performance, and the Brier Score in the calibration curve was 0.210.

**Conclusion:**

This study provided a predictive model for formulating individualized diagnostic strategies of suspected FIPAP infants. However, due to the limitations of the lack of prospective dataset validation, future studies should further validate the clinical application potential of the predictive model to improve the diagnostic efficiency and quality of life of FPIAP.

## 1 Introduction

The prevalence of gastrointestinal disorders resulting from food allergies in infants has been on the rise, particularly in developing nations (1). These conditions can be classified into three types based on their clinical manifestations and involvement of the gastrointestinal tract including food protein-induced allergic proctocolitis (FPIAP), food protein-induced enteropathy (FPIE), and food protein-induced enterocolitis syndrome (FPIES) (2). Among breastfed infants, FPIAP is the most prevalent clinical subtype characterized by the presence of bloody stool with or without diarrhea and mucus (3, 4). It involves chronic inflammation of the distal sigmoid colon and rectum, eliciting a cell-mediated response that leads to mucosal edema, erosions and ulcerations (5, 6).

Lactating mothers consume diverse diets that include multiple food allergens. While cow's milk is the primary causative protein, soy, egg, and wheat may also be implicated (7). Additionally, if the infant has risk factors such as altered intestinal permeability or an incomplete establishment of intestinal flora, the ingestion of breast milk containing allergens can lead to gastrointestinal allergic reactions (8, 9). The elimination of allergenic foods, particularly cow's milk, from the mother's diet typically leads to symptom remission in most cases. However, unnecessary strict dietary restrictions may have detrimental effects on the lactation process in breastfeeding mothers. Moreover, in cases where maternal dietary elimination proves ineffective in alleviating symptoms, consideration should be given to discontinuing breastfeeding and transitioning to the hypoallergenic formula (10). Therefore, the provision of accurate guidance on breastfeeding is an essential component in the nutritional management of infants diagnosed with FPIAP.

Although clinical management is conducted in accordance with clinical guidelines and consensus, the real-world implementation varies due to individual immune responses, clinical characteristics, diverse feeding patterns, family economic conditions, and the compliance of mothers and family members. As a result, breastfeeding persistence for FPIAP infants may be compromised. Therefore, finding a balance between breastfeeding and the use of hypoallergenic formula to alleviate symptoms while ensuring continuity of breastfeeding has become a crucial clinical concern.

Ethical considerations and subject protection present challenges to conducting clinical research in special populations, particularly infants. Establishing disease-specific cohorts and conducting real-world studies are important approaches to promote breastfeeding among infants with diseases, especially those experiencing feeding problems. Breastfeeding is the optimal source of nutrition for all infants, including those with FPIAP. The objective of this study was to investigate the factors associated with the results of maternal dietary avoidance in infants diagnosed with Food Protein-Induced Allergic Proctocolitis (FPIAP). Additionally, we aimed to develop a predictive model using machine learning techniques to forecast the results of maternal dietary restrictions. This could provide a foundation for formulating more evidence-based treatment plans for FPIAP, enhancing the sustained breastfeeding rates among affected infants, and optimizing the utilization of hypoallergenic formula.

## 2 Materials and methods

### 2.1 Patients

Infants diagnosed with FPIAP in our center, the tertiary children's hospitals, during 2020 1st J January and 1st September 2023 were enrolled retrospectively. Patients were eligible for inclusion if they met the following criteria: (1) aged between 1 and 12 months; (2) predominantly breastfed (infant formula <20% or ≤2 times/day) when they were diagnosis of FPIAP; (3) bloody stool as the primary manifestation; (4) follow up persistently for 12 months at our hospital with complete record of the diagnosis and treatment issues. If patients had a history of neonatal enterocolitis syndrome, gastrointestinal surgery, or coexisting gastrointestinal disorders, they were excluded from the study cohort.

### 2.2 Study design

Infants suspected of FPIAP based on their medical history necessitated a 2-weeks period for diagnostic allergen elimination through maternal dietary restriction or the utilization of hypoallergenic formulas including AAF or eHF determined by doctors based on the severity. If symptoms were resolved after dietary elimination, they were considered as “effective”. Subsequently, open oral food challenges (OFCs) were conducted in accordance with clinical guidelines (11). In the event of symptom recurrence resulting in positive outcomes, a diagnosis was established. After the diagnosis was established, the patients were fed according to the effective way of dietary elimination pattern for six months.

The clinical data, including demographic information, clinical characteristics, medication history, family history, diagnosis and treatment details, as well as laboratory test results were extracted from Hospital Information Manage System and Laboratory Information System for statistical analysis. The presence of gross bloody stools with positive occult blood or 5–10/hp of red blood cells on microscopic examination was defined as mild, while the condition was classified as moderate if the number of red blood cells exceeded 10/hp. Eczema area and severity index (EASI) was used to assess the severity of eczema (12). The EASI Score ranging from 1.1 to 7.0 was categorized as mild, while a score between 7.1 and 21.0 was classified as moderate.

The FPIAP infants were divided into two groups based on the results of maternal dietary restriction, and an analysis was conducted to identify the factors influencing the results of diagnostic maternal dietary restriction. This study was conducted in accordance with the approved protocol by the Ethics Committee of Hospital.

### 2.3 Statistical analysis

This study is a quantitative study, and all Statistical analyses were performed using R version 4.2.3 and python version 3.11.4. Categorical data were presented as numerical values and percentages, while normally distributed continuous variables were expressed as means ± standard deviations (SD). Non-normally distributed variables were represented by medians and interquartile ranges [IQR]. The comparison between the two groups for categorical variables was conducted using the *χ*2 test or Fisher's exact test. For continuous variables that deviated from normal distribution, the Mann–Whitney-U nonparametric test was employed for comparison. Univariate analysis was performed using a binary logistic regression model. LassoCV, logistics regression, XGBoost, and Random Forest were used to rank the importance of variables. Then the final variable selection was carried out using Lasso regression model. Classification models were built utilizing various machine learning algorithms including XGB Classifier, Logistic Regression, Random Forest Classifier, Ada Boost Classifier, KNeighbors Classifier, LGBM Classifier, Decision Tree Classifier, Gradient Boosting Classifier, Support Vector Classifier. The random seed was set to 42, the proportion of the validation set was 0.2, and a 10-fold cross-validation were performed. The indicators of the training set and the validation set were compared including AUC (Area Under Curve), specificity, sensitivity, accuracy, positive predictive value (PPV), negative predictive value (NPV) and F1 score between the training set and validation set. The optimal algorithm was selected to construct the final prediction model. The logistic regression model was visualized using nomogram, calibration curve was provided, and Brier Score was calculated. Hosmer and Lemeshow goodness of fit test method was used to evaluate the fitting degree of the logistic regression model. SHAP performed model interpretation, provided feature importance ranking and summary plot according to SHAP value of factors. The difference was statistically significant as *P* < 0.05.

## 3 Results

### 3.1 Clinical characteristics of enrolled FPIAP infants

A total of 693 cases meeting the inclusion criteria with complete data elements were included in the study. The median age was 73.000[40.000,116.000] days, with 385 males and 308 females. Majority of cases (445/693,64.21%) had a disease course within 2 weeks. Mild hematochezia was observed in most cases, while moderate to severe hematochezia was present in 53 cases (7.65%). Additionally, mucus in the stool was reported by 46.90% (325/693) of the cases. Skin symptoms were found in 35.93% (249/693) of infants, including moderate eczema in 42 cases. Respiratory tract symptoms were observed in 8.08% (56/693) of infants, and malnutrition was noted in 60 (8.66%) infants as well. A total of 164 cases (23.67%) experienced hematochezia in neonatal period, among which 51% had a history of antibiotic use. Comparing FPIAP infants fed with partial breastfeeding to those exclusively breastfed revealed that regurgitation (*χ*2 = 8.807, *P* = 0·003), respiratory symptoms (*χ*2 = 4.654, *P* = 0·031), and increased peripheral blood eosinophils (*χ*2 = 22.333, *P* = 0·0003) were more commonly seen.

### 3.2 Comparison of symptoms remission among different diagnostic dietary elimination patterns

**Figure 1:**
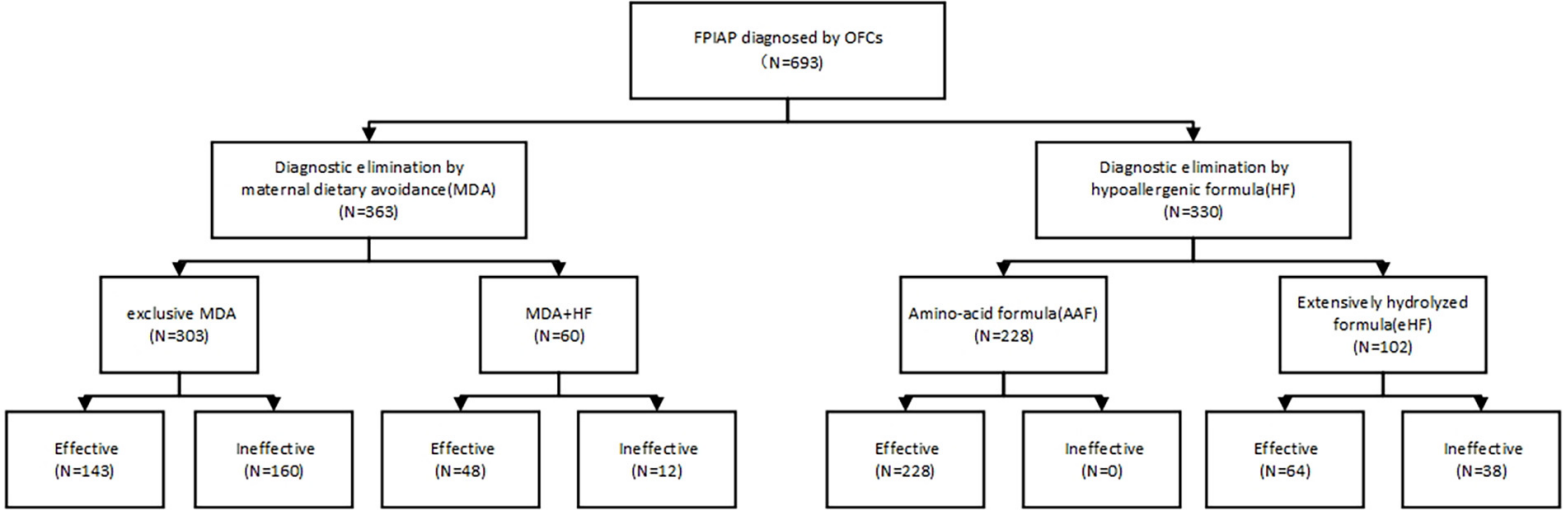
Groups.

The total remission rate of symptoms through maternal dietary restriction was 47.38% (172/363). The symptoms remission rates for diagnostic maternal dietary elimination in combination with eHF and AAF were 35% (21/60) and 44.44% (27/60), respectively, showing no significant difference (*χ*2 = 0.404, *P* = 0.525). The overall efficacy of hypoallergenic formula was 88.48% (292/330), with AAF and eHF achieving rates of 100% (228/228) and 62.75% (64/102) respectively (Figure 1). Patients who failed to maternal dietary avoidance or eHF and switched to AAF could effectively relieve clinical symptoms. There were statistically significant differences in symptom remission rates among maternal dietary elimination, hypoallergenic formula, and the combination of both approaches (*χ*2 = 27.417, *P* < 0.001).

### 3.3 The influencing factors of diagnostic maternal dietary elimination results

The factors that might impact the results of maternal dietary elimination were found to have statistically significant differences between groups, including age, disease duration, regurgitation, eczema, neonatal hematochezia (Table 1). Univariate analysis results suggested that the factors including age, disease duration, regurgitation, eczema, history of neonatal hematochezia were different between groups with statistical significance.

**Table 1:** Influencing factors of the diagnostic maternal dietary elimination results.

Factors	Diagnostic Maternal dietary restriction		U/*Χ*^2^	*P*
	Effective (*n* = 172)	Ineffective (*n* = 191)		
Age onset, median[IQR]	89.000[53.000,126.000]	75.000[41.000,111.000]	−2.481	0.013
Gender, *n* (%)				
Male	96 (55.814)	96 (50.262)	1.120	0.290
Female	76 (44.186)	95 (49.738)		
Duration, *n* (%)				
<2 weeks	127 (73.837)	109 (57.068)	11.189	0.004
2–4 weeks	27 (15.698)	49 (25.654)		
>4 weeks	18 (10.465)	33 (17.277)		
Diarrhea, *n* (%)				
No	91 (52.907)	88 (46.073)	0.038	0.846
Yes	81 (47.093)	103 (53.927)		
Stool frequency, *n* (%)				
<5 times/d	110 (63.953)	119 (62.304)	0.109	0.947
5–10 times/d	57 (33.140)	66 (34.555)		
>10 times/d	5 (2.907)	6 (3.141)		
Bloody stools, *n* (%)				
Mild	163 (94.767)	176 (92.147)	1.007	0.316
Moderate	9 (5.233)	15 (7.853)		
Stool mucus, *n* (%)				
No	89 (51.744)	85 (44.503)	1.902	0.168
Yes	83 (48.256)	106 (55.497)		
Regurgitation, *n* (%)				
No	158 (91.860)	161 (84.293)	4.865	0.027
Yes	14 (8.140)	30 (15.707)		
Eczema, *n* (%)				
No	113 (65.698)	102 (53.403)	6.917	0.031
Mild	53 (30.814)	74 (38.743)		
Moderate	6 (3.488)	15 (7.853)		
Respiratory symptoms, *n* (%)				
No	164 (95.349)	173 (90.576)	3.101	0.078
Yes	8 (4.651)	18 (9.424)		
Malnutrition, *n* (%)				
No	159 (92.442)	180 (94.241)	0.474	0.491
Yes	13 (7.558)	11 (5.759)		
Recent antibiotics usage, *n* (%)				
No	141 (81.977)	151 (79.058)	0.490	0.484
Yes	31 (18.023)	40 (20.942)		
Neonatal antibiotics usage, *n* (%)				
No	161 (93.605)	168 (87.958)	3.399	0.065
Yes	11 (6.395)	23 (12.042)		
Neonatal hematochezia, *n* (%)				
No	152 (88.372)	150 (78.534)	6.265	0.012
Yes	20 (11.628)	41 (21.466)		
Poor intake, *n* (%)				
No	152 (88.372)	173 (90.576)	0.469	0.493
Yes	20 (11.628)	18 (9.424)		
Preterm, *n* (%)				
No	164 (95.349)	183 (95.812)	0.046	0.830
Yes	8 (4.651)	8 (4.188)		
Family allergic disease history, *n* (%)				
No	144 (83.721)	153 (80.105)	0.796	0.372
Yes	28 (16.279)	38 (19.895)		
Increase peripheral EOS ratio, *n* (%)				
No	113 (65.698)	108 (56.545)	3.234	0.198
< twice	28 (16.279)	41 (21.466)		
>twice	31 (18.023)	42 (21.990)		
Anemia, *n* (%)				
No	157 (91.279)	175 (91.623)	0.014	0.907
Yes	15 (8.721)	16 (8.377)		
PLT, *n* (%)				
Normal	153 (88.953)	165 (86.387)	0.549	0.459
Increase	19 (11.047)	26 (13.613)		
Intestinal infection, *n* (%)				
No	150 (87.209)	163 (85.340)	0.266	0.606
Yes	22 (12.791)	28 (14.660)		
Suspected allergen ingestion, *n* (%)				
No	152 (88.372)	179 (93.717)	3.217	0.073
Yes	20 (11.628)	12 (6.283)		
Feeding pattern, *n* (%)				
Exclusive BF	152 (88.372)	167 (87.435)	0.075	0.785
Predominate BF	20 (11.628)	24 (12.565)		
Maternal dietary elimination time, *n* (%)				
≤2 weeks	157(91.279)	183(95.812)	3.133	0.077
>2 weeks	15(8.721)	8(4.188)		

### 3.4 Predictive models for maternal dietary avoidance outcomes

**Figure 2:**
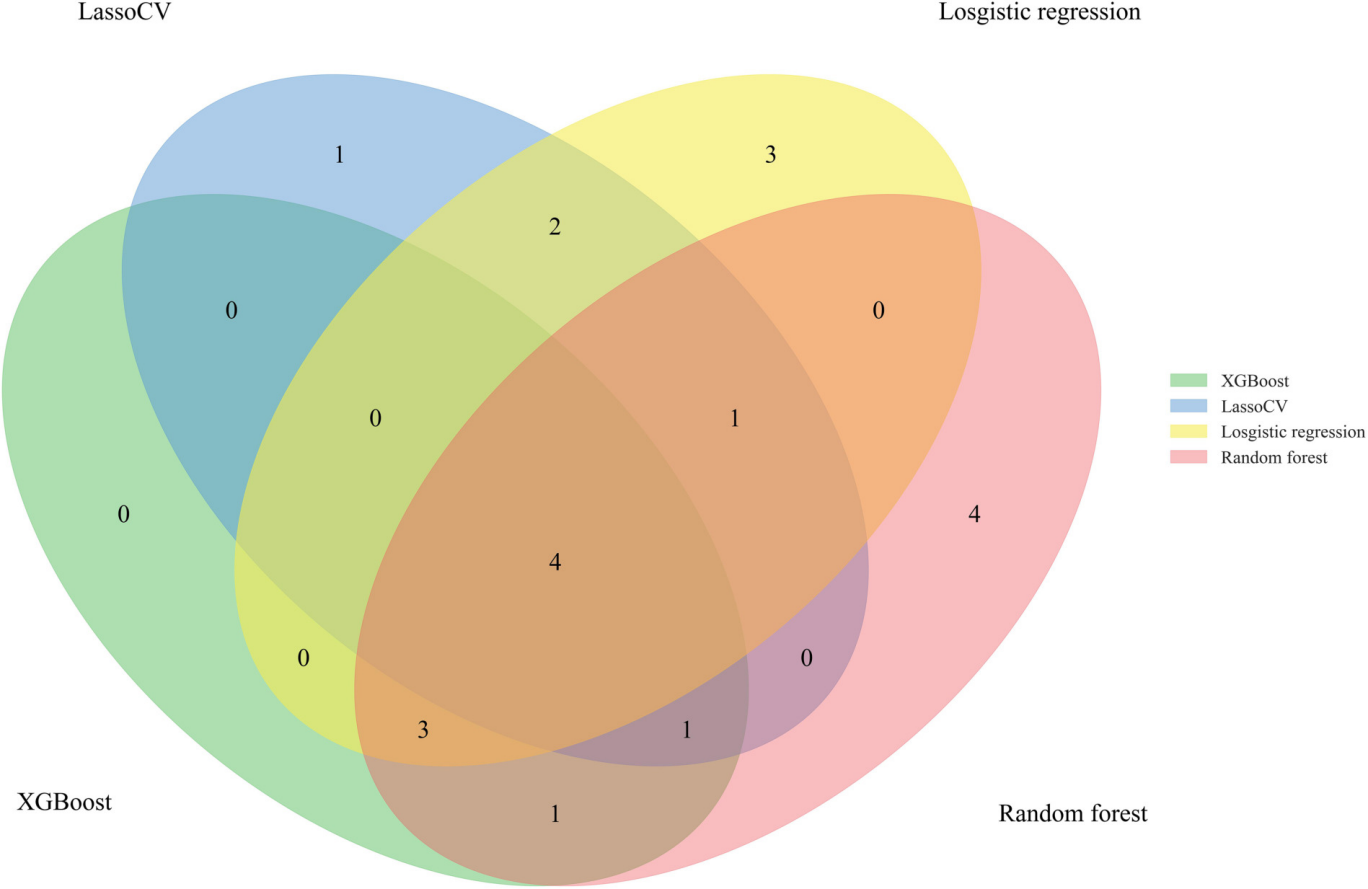
Venn diagram.

**Figure 3:**
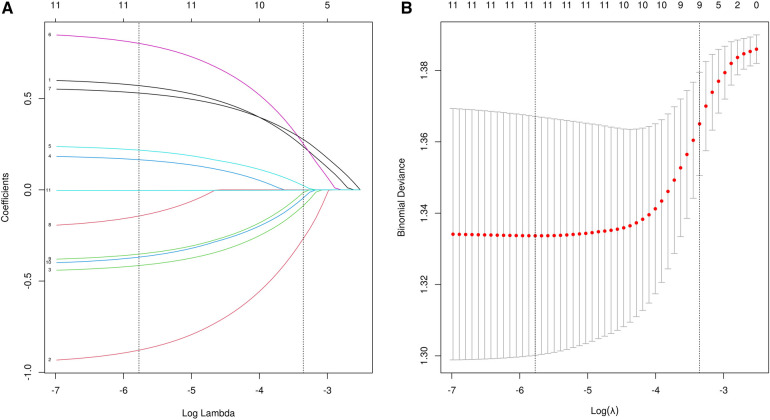
**(A)** Profile of LASSO coefficients. **(B)** Lasso regression cross-validation curves.

A total of 24 variables were included. Firstly, the importance of the variables was ranked, and then the intersection was taken to screen out 4 variables, including diarrhea, eczema, EOS, mucus, shown as Venn diagram (Figure 2). Meanwhile, five variables were added after discussion according to clinical experience and significance including regurgitation, duration, maternal dietary elimination time (MDET), age, mother suspected allergen ingestion (MSAI), feeding pattern (FP), Neonatal hematochezia (NH). LASSO regression was used to determine the final variables, with the standard error of the minimum distance *λ*=0.035 (Figure 3A), and 9 variables corresponding to the model were selected (Figure 3B): eczema, MSAI, mucus, EOS, regurgitation, duration, diarrhea, MDET, age.

**Figure 4:**
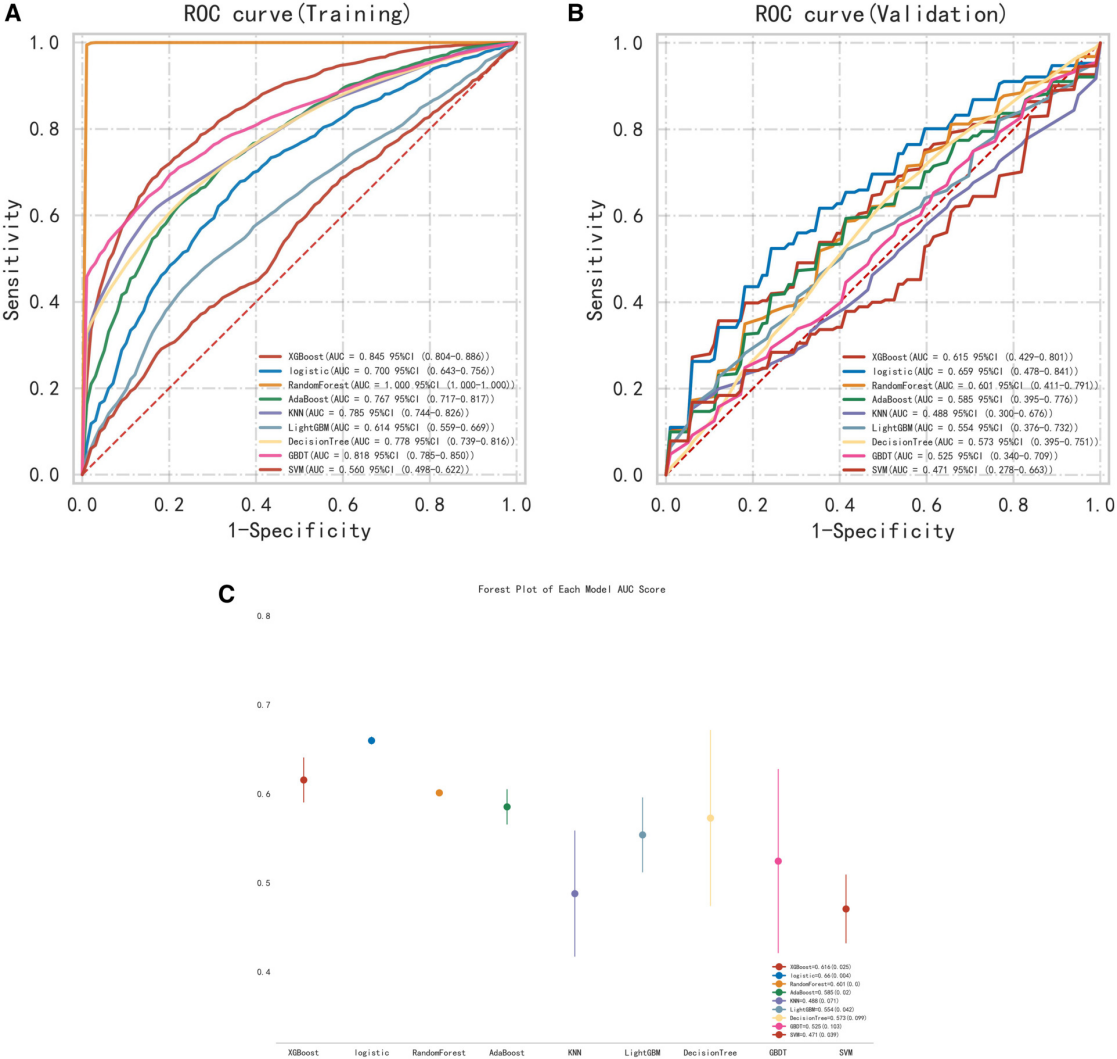
**(A)** ROC curve of the training set. **(B)** ROC curve of the validation set. **(C)** Multi-model forest plot of the validation set.

The 9 selected variables were entered into the construction of multiple machine learning models. Including XGB Classifier, Logistic Regression, Random Forest Classifier, Ada Boost Classifier, KNeighbors Classifier, LGBM Classifier, Decision Tree Classifier, Gradient Boosting Classifier, and Support Vector Classifier were used to determine the best model (Figures 4A,B). According to the AUC ranking, the best performance of the training set was Random Forest, and the best performance of the validation set was logistic regression (Figure 4C). The Random Forest had overfitting phenomenon, and the logistic regression model had relatively good relative stability. Therefore, logistic regression was finally selected for modeling.

**Figure 5:**
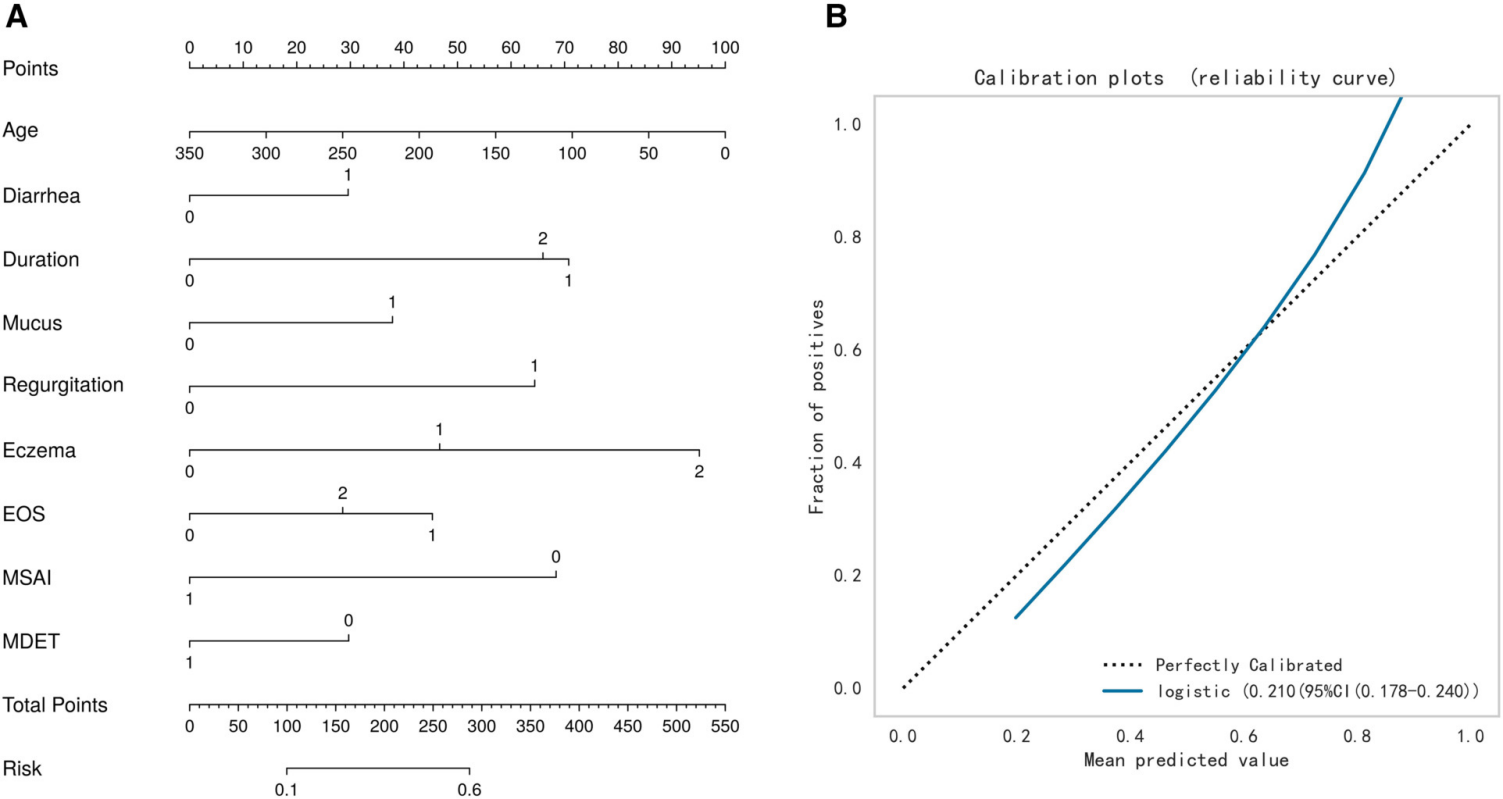
**(A)** Nomogram. **(B)** Calibration curve.

The test set was randomly selected from the total sample, and the number of samples was 72 (20.00%). The remaining samples were used as the training set for 10-fold cross validation, and the AUC was 0.657(95%CI: 0.453–0.858) in the validation set. The AUC of the final model in the test set was 0.743, and the accuracy was 0.699 (Table 2). Under the AUC indicator, the validation set exceeded the test set by less than 10%, and the Hosmer and Lemeshow goodness of fit test method was used to evaluate the fitting degree of the logistic regression model, and *P* = 0.691 > 0.05 was considered to be successful. Finally, nomorgram diagram was used to visualize the model performance (Figure 5A), and the Brier Score in the calibration curve was 0.210 (Figure 5B).

**Table 2:** Performance indicators of logistic regression model in different data sets.

Data set	AUC (95%CI)	Cut-off (95%CI)	Accuracy (95%CI)	Sensitivity (95%CI)	Specificity (95%CI)	PPV (95%CI)	NPV (95%CI)	F1 score (95%CI)
Training set	0.690 (0.626–0.754)	0.525 (0.510–0.539)	0.651 (0.642–0.661)	0.634 (0.599–0.668)	0.671 (0.637–0.704)	0.678 (0.663–0.693)	0.629 (0.615–0.643)	0.653 (0.637–0.669)
Validation set	0.657 (0.453–0.858)	0.525 (0.510–0.539)	0.603 (0.539–0.668)	0.588 (0.500–0.675)	0.619 (0.569–0.669)	0.620 (0.558–0.682)	0.590 (0.521–0.659)	0.602 (0.528–0.676)
Testing set	0.743 (0.626–0.860)	0.511	0.699	0.75	0.636	0.714	0.677	0.732

**Figure 6:**
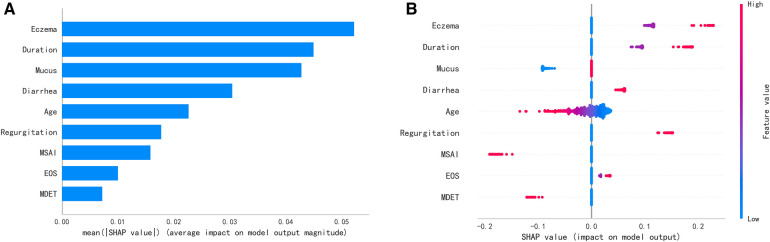
**(A)** Feature importance. **(B)** Summary plot.

SHAP explained the ranking of the contribution of variables in the model to the prediction model (Figure 6A). It is worth noting that the summary plot showed that more severe eczema, longer course of eczema, and reflux contributed more to the prediction of ineffective result of maternal dietary avoidance. On the contrary, the mother's suspicious allergen intake history contributed more to the prediction of effective result of maternal dietary avoidance. Younger age was associated with a more pronounced contribution to the prediction of maternal dietary avoidance ineffectiveness (Figure 6B).

## 4 Discussion

This study focused on food protein-induced proctocolitis (FPIAP), a non-IgE mediated food allergy commonly seen in infants and young children, which is mainly manifested by mucous and bloody stool (13, 14). It is one of the infant intestinal diseases with increasing incidence at present, and can affect growth and development as well as long-term intestinal health in severe cases. Although the true prevalence of cow's milk allergy (CMA) remains controversy, there has been an observed increase in the incidence rate of haematochezia in infants, which was commonly attributed to FPIAP, the most prevalent non-IgE mediated food allergic gastrointestinal disorder. In this study cohort, neonatal hematochezia warrants attention in clinical features. There are multiple etiologies associated with bloody stools in newborns. In recent years, an increasing number of disease cohort studies have reported cases of neonatal hematochezia attributed to intestinal allergies (15–18). The immaturity of the neonatal intestinal barrier can result in compromised integrity, heightened permeability, impaired food protein macromolecule degradation, and facilitated translocation across the gut epithelium, thereby predisposing to allergic reactions (9). In our cohort, 164 cases exhibited hematochezia during the neonatal period, with 51 of them (31.10%) having a documented history of antibiotic usage in this period. It can be inferred that the establishment and colonization of neonatal intestinal microbiota play a crucial role in relation to early postnatal hematochezia; however, further investigation is required to elucidate the specific pathogenesis (19, 20).

The management of most infants with FPIAP typically involves a three-step approach: a diagnostic elimination diet, followed by home reintroduction for challenge and long-term implementation of a therapeutic elimination diet. Despite strict adherence to maternal dietary avoidance, inadequate clinical relief may still occur due to suboptimal elimination or the presence of cross-reactive allergenic foods (21). The precise diagnosis continues to pose a challenge due to the possibility of both under- and over-diagnosis, resulting in adverse health consequences and financial burdens. Therefore, it is important to comprehend the influential factors that impact the outcomes of maternal dietary elimination in order to establish an accurate diagnosis, with the aim of preventing nipple confusion or refusal of feeding bottles by breastfed infants, as well as their inability to accept the taste of hypoallergenic formula. Our study found that age, disease duration, regurgitation, eczema, history of neonatal hematochezia were the risk factors contributing to the ineffectiveness of maternal dietary elimination, while the history of suspicious allergen ingestion provided by the mothers was the protective factor. Meanwhile, we have summarized and compared real-world elimination therapy strategies, finding that the application of AAF demonstrated the highest effectiveness rate, followed by eHF. This indicates that hypoallergenic formula has advantages over maternal dietary elimination in terms of allergy diagnosis due to its low allergenicity, which leads to improved diagnostic efficiency. Molnar et al. reported on a cohort of 30 children with FPIAP. Within this cohort, maternal dietary elimination resulted in improvement for only 8 out of the 30 children, while the remaining 22 did not show any improvement. However, administration of an AAF led to complete resolution of rectal bleeding (22). Barni et al. also reported that 18% of their patients required to switch to an amino acid formula (23). Recent health economic studies suggest that choosing AAF as a first-line option can result in cost savings and reduced overall administrative expenses in non-breast-fed infants (24). In general, combining patient conditions and relevant factors that influence maternal dietary elimination effects can facilitate the selection of a more appropriate feeding pattern, thereby enhancing diagnostic efficiency.

However, this study also has some limitations, mainly reflected in the single-center study design and the lack of validation of prospective data sets. Although we used machine learning algorithms to build predictive models and rigorous data analysis methods, the diversity of the sample and the limitations of the clinical context may affect the universality of the results. Nevertheless, our study did not include the psychosocial and environmental factors associated with the mother dietary restriction, such as the impact of dietary elimination on maternal and familial quality of life (25), as well as maternal willingness to sustain breastfeeding. In the real world, it was evident that many parents experience heightened concerns and anxiety when confronted with the presence of blood in their infants’ stool. This occurrence can lead to significant distress for parents (26). Consequently, if hematochezia recurs or fails to improve within the elimination period, some mothers may proactively choose to discontinue breastfeeding. In this study, the suspected allergens consumed by mothers were primarily focused on milk and food allergen, but did not include medications used by mothers (which may contain allergenic components). Therefore, future studies should consider the factor of medications used by mothers. In addition, there are limit researches in this specific area. Future studies should prioritize assessing the additional burden associated with dietary adherence and the impact on the quality of life of mothers and families, including the use of psychological scales or quality of life scores, to gain a more comprehensive understanding of the factors influencing maternal dietary avoidance and to incorporate models that ultimately improve their accuracy and predictive power.

## 5 Conclusion

Retrospective data analysis with a certain sample size was conducted to understand the clinical characteristics of infants with FPIAP and the influencing factors of different diagnostic protocols. On this basis, we constructed a predictive model by multiple machine learning algorithm to predict the outcome of mothers’ dietary avoidance, which provides a tool for selecting a reasonable diagnostic approach for the suspected diagnosis of FPIAP in breastfeeding infants. However, due to the limitations of the lack of prospective dataset validation, future studies should further validate the clinical application potential of the predictive model to improve the diagnostic efficiency and quality of life of FPIAP.

## Data Availability

The datasets presented in this study can be found in the FigShare repository at DOI: 10.6084/m9.figshare.28253834.
